# Triglyceride–glucose index and structural chromosome aberrations in medical radiation workers: a cross-sectional study with a stratified dosimetry substudy

**DOI:** 10.3389/fpubh.2026.1908697

**Published:** 2026-09-14

**Authors:** Lingying Fan, Lu Wang, Qiurong He, Yufan Tang, Yiping Chen

**Affiliations:** West China School of Public Health and West China Fourth Hospital, Sichuan University, Chengdu, Sichuan, China

**Keywords:** chromosome aberrations, insulin resistance, medical radiation workers, occupational dosimetry, occupational health, triglyceride–glucose index

## Abstract

**Background:**

Metabolic status may be associated with variation in cytogenetic outcomes among occupationally exposed workers. We examined the cross-sectional association between the triglyceride–glucose (TyG) index and structural chromosome aberration burden among medical radiation workers.

**Methods:**

This retrospective cross-sectional study included 23,408 medical radiation workers and 6,542 healthcare controls without occupational radiation duties. The primary outcome was the number of structural chromosome aberrations per 100 metaphases, analyzed using negative binomial regression. A stratified dosimetry substudy of 1,500 medical radiation workers incorporated recorded cumulative occupational dose, with inverse-probability weighting used as a sensitivity analysis for unequal sampling fractions across occupational categories.

**Results:**

Among medical radiation workers, each 1-SD increase in TyG was associated with a higher structural chromosome aberration count after multivariable adjustment (adjusted IRR, 1.15; 95% CI, 1.09–1.22). Compared with the lowest TyG quartile, the adjusted IRR was 1.52 (95% CI, 1.27–1.83) for the highest quartile (*P* for trend < 0.001). Restricted cubic spline analysis suggested a non-linear pattern, with a steeper estimated association at higher TyG values (*P* for non-linearity = 0.013). In the dosimetry substudy, the adjusted IRR was 1.27 (95% CI, 1.14–1.42) per 1-SD increase after adjustment for recorded cumulative occupational dose and other covariates; the inverse-probability-weighted estimate was similar (IRR, 1.25; 95% CI, 1.11–1.41). Recorded cumulative occupational dose was also positively associated with structural chromosome aberration count after additional adjustment for TyG (adjusted IRR per 10-mSv increase, 1.10; 95% CI, 1.04–1.17).

**Conclusions:**

Higher TyG was associated with a greater structural chromosome aberration count among medical radiation workers, including after adjustment for recorded cumulative occupational dose. The cross-sectional design, incomplete exposure characterization, and potential residual confounding preclude causal interpretation or use of TyG for individual occupational risk stratification.

## Highlights

- What is already known: Structural chromosome aberrations are established cytogenetic indicators of biological effect in radiation protection, but the extent to which metabolic status is associated with variation in aberration burden among medical radiation workers remains unclear.
- What this study adds: In a large cross-sectional population of medical radiation workers, a higher triglyceride–glucose index was associated with a greater structural chromosome aberration count. The association remained evident in a stratified dosimetry substudy after adjustment for recorded cumulative occupational dose and was similar after inverse-probability weighting.
- How this study might affect research or practice: The findings support further prospective investigation of metabolic health in relation to cytogenetic outcomes but do not establish causality, temporal order, or individual radiosensitivity. The TyG index should not currently be used to estimate radiation dose, modify occupational dose limits, or stratify individual occupational risk.

## 1 Introduction

The expanding use of ionizing radiation in medical diagnosis and treatment has increased the number of healthcare personnel who experience repeated or prolonged occupational exposure, generally at low doses (1, 2). Personal dosimetry remains the foundation of occupational radiation protection, but recorded external dose may not fully capture all relevant exposure characteristics or explain variation in cytogenetic outcomes among workers with similar recorded doses (3–6). Structural chromosome aberrations in peripheral blood lymphocytes can result from DNA double-strand breaks and their misrepair and are established cytogenetic indicators used in radiation protection and biological dosimetry (7). Higher frequencies of chromosome aberrations and micronuclei have also been prospectively associated with subsequent cancer risk at the population level (8, 9). Cytogenetic assessment may therefore complement physical dosimetry by providing information on biological effect, although it is not a substitute for exposure measurement or an individual predictor of future disease.

Differences in chromosome aberration frequency among workers with apparently similar occupational histories may reflect variation in recorded exposure, exposure not captured by routine monitoring, lifestyle factors, co-exposures, and host biological characteristics (10–12). Metabolic status may be relevant in this context. Insulin resistance and related metabolic disturbances are associated with oxidative stress and chronic low-grade inflammation (13–15), and metabolic signaling is linked to cellular DNA damage-response and repair pathways (16–19). These processes provide a plausible basis for an association between metabolic dysfunction and structural chromosome aberrations. However, epidemiological evidence directly evaluating this relationship in occupationally exposed populations remains limited.

The triglyceride–glucose (TyG) index, calculated from fasting triglyceride and fasting glucose concentrations, is a practical surrogate marker of insulin resistance that has been evaluated against the euglycemic–hyperinsulinemic clamp (20, 21). Because both components are routinely measured during occupational health examinations, TyG can be derived without additional specimen collection or specialized testing. Previous studies have focused primarily on associations of TyG and related insulin-resistance indices with cardiovascular and other cardiometabolic outcomes (22–25). It remains unclear whether TyG is associated with structural chromosome aberration burden among medical radiation workers and whether any such association persists after accounting for recorded cumulative occupational dose and conventional covariates.

We therefore conducted a retrospective cross-sectional study of medical radiation workers, with healthcare personnel without occupational radiation duties included as a secondary comparison group. The primary objective was to evaluate the association between TyG and structural chromosome aberration burden among medical radiation workers. A stratified dosimetry substudy was used to evaluate this association after accounting for recorded cumulative occupational dose, to directly examine the dose–aberration association, and to explore chromosome-type and chromatid-type aberrations separately. We also examined whether the TyG-associated aberration count differed according to occupational radiation-work status.

## 2 Materials and methods

### 2.1 Study design and population

This retrospective cross-sectional study used occupational health surveillance and routine health-examination data collected at West China Fourth Hospital, Sichuan University, between February 2022 and September 2024. Eligible participants were adults aged ≥18 years with fasting glucose, triglyceride, cytogenetic outcome, and prespecified covariate data obtained at the same health examination. Individuals with malignancy or hematological disease, previous chemotherapy or radiotherapy, or pregnancy were excluded. Each participant contributed one examination; for participants with multiple eligible examinations, the latest eligible examination was retained. Analyses were restricted to participants with complete data, and missing values were not imputed.

Medical radiation workers undergoing occupational health surveillance constituted the primary analytical population. Healthcare controls were personnel without documented occupational radiation duties who participated in a standardized institutional health-examination program during the same period. Cytogenetic assessment was included as a uniform component of the examination program and was not initiated because of suspected chromosome abnormalities or related clinical symptoms. Cytogenetic testing for both groups was performed through the same institutional laboratory workflow under contemporaneous standard operating procedures. The same eligibility and exclusion criteria were applied to both groups. Controls were sampled without replacement within prespecified age-by-sex strata to approximate the age and sex distribution of the medical radiation-worker population. This represented group-level stratified sampling rather than individual matching. The final analytical population included 23,408 medical radiation workers and 6,542 healthcare controls. Further details of control sampling are provided in the Supplementary Methods.

The study was approved by the Institutional Review Board of West China Fourth Hospital, Sichuan University (HXSY-EC-2025002). The requirement for informed consent was waived because de-identified retrospective data were used.

### 2.2 Covariates and TyG index

Demographic characteristics, anthropometric measurements, current smoking, and current alcohol consumption were obtained from electronic health records. Smoking and alcohol-consumption status were analyzed according to the binary status recorded at the index health examination. BMI was calculated as weight in kilograms divided by height in meters squared. For medical radiation workers, occupational category and duration of radiation-related employment were obtained from the occupational health registry. Occupational categories were classified as diagnostic radiology, nuclear medicine, and interventional radiology.

Fasting venous blood for biochemical testing and peripheral blood for cytogenetic assessment were collected on the same day. Fasting glucose and triglycerides were measured enzymatically using a Roche Cobas c701 analyzer. Concentrations were converted from mmol/L to mg/dL using conversion factors of 18.02 for glucose and 88.57 for triglycerides.

The TyG index was calculated as: *TyG index* = *ln [fasting triglycerides (mg/dL)* × *fasting glucose (mg/dL) / 2]*. TyG was analyzed primarily as a continuous variable per 1-SD increase, with quartiles used as a complementary parameterization.

### 2.3 Chromosome aberration assessment

The primary cytogenetic outcome was the total number of independently scored structural chromosome aberration events per 100 eligible metaphases. Operationally, total structural chromosome aberration count was defined as the sum of chromosome-type and chromatid-type events. Chromosome-type events comprised dicentric chromosomes and centric rings. Chromatid-type events comprised chromatid breaks and chromatid exchanges, including radial configurations. Chromatid gaps and isolated acentric fragments were excluded. An acentric fragment accompanying a dicentric chromosome or centric ring was recorded as part of the corresponding event and was not counted as an additional independent event. Reciprocal translocations and inversions were not comprehensively detectable using the conventional Giemsa-based assay and were therefore not analyzed as separate outcomes.

Exactly 100 eligible metaphases were evaluated for each participant; therefore, the outcome represented the number of structural chromosome aberration events per 100 metaphases. A participant with more than one independently scored aberration contributed multiple events to the count outcome but was counted only once in analyses describing the number of participants with at least one aberration.

In the full analytical population, the standardized institutional cytogenetic database retained the aggregate structural aberration count per 100 metaphases based on the same event definitions but did not consistently preserve event-level subtype labels. Consequently, subtype-specific analyses were restricted to the dosimetry substudy, in which archived chromosome preparations were re-evaluated. In the dosimetry substudy, archived preparations were independently re-evaluated by two experienced cytogeneticists blinded to TyG values, occupational categories, and recorded cumulative occupational dose. Discrepancies were resolved by joint review. Detailed culture, slide-preparation, metaphase-selection, scoring, and quality-control procedures are provided in the Supplementary Methods.

### 2.4 Stratified dosimetry substudy

A stratified dosimetry substudy was conducted within the medical radiation-worker population. Eligibility required an uninterrupted sequence of available quarterly personal dosimetry records from the first valid record to the last completed monitoring quarter before cytogenetic sampling, together with archived chromosome preparations suitable for re-evaluation. The eligible source population comprised 8,420 diagnostic radiology workers, 2,680 nuclear medicine workers, and 3,150 interventional radiology workers.

Within each occupational category, 500 participants were selected by computer-generated simple random sampling, yielding 1,500 participants. Selection within each category was independent of TyG, recorded cumulative occupational dose, and chromosome aberration status. Because equal allocation differed from the occupational-category distribution of the eligible source population, inverse-probability weighting was used in a sensitivity analysis to standardize the substudy to that distribution.

Occupational external radiation exposure was monitored quarterly using personal thermoluminescent or optically stimulated luminescence dosimeters supplied and processed by certified occupational radiation monitoring agencies. Dosimeters were exchanged quarterly, and each reported value represented the dose accumulated over the corresponding three-month monitoring period. Recorded cumulative occupational dose was defined as the sum of all valid agency-reported quarterly dose values from the first available valid record to the last completed monitoring quarter before cytogenetic sampling. Workers with one or more missing quarterly records within this observation window were not eligible for the substudy. The available monitoring interval did not necessarily include exposure accrued before the first available record. Recorded cumulative occupational dose was modeled continuously, and dose-associated estimates were reported per 10-mSv increase. Additional details are provided in the Supplementary Methods.

### 2.5 Statistical analysis

Continuous variables are presented as mean ± SD or median (IQR), as appropriate, and categorical variables as *n* (%). Absolute standardized mean differences (SMDs) were used descriptively to quantify between-group differences and to assess covariate balance after propensity score matching. For continuous variables, the absolute SMD was calculated as the absolute difference in means divided by the pooled SD; for binary variables, the absolute difference in proportions was divided by the pooled binomial SD. SMDs are descriptive measures rather than hypothesis-test *P*-values. An absolute SMD < 0.10 was conventionally interpreted as a small between-group difference and, in the matched analysis, as indicating adequate covariate balance.

The structural chromosome aberration count per 100 metaphases was the primary outcome. The variance of the count exceeded its mean, indicating overdispersion; therefore, negative binomial regression was used. Exponentiated regression coefficients are reported as incidence rate ratios (IRRs) by model convention and represent ratios of expected structural chromosome aberration counts per 100 evaluated metaphases; they do not represent incidence rates over person-time. No offset was included because exactly 100 metaphases were evaluated for each participant. The complementary binary outcome, defined as the presence of at least one structural chromosome aberration, was analyzed using logistic regression, with results reported as odds ratios (ORs) and 95% CIs.

The primary analysis evaluated continuous TyG per 1-SD increase in relation to structural chromosome aberration count among medical radiation workers. Complementary analyses modeled TyG in quartiles and evaluated the binary outcome. Continuous and quartile-based TyG terms were entered into separate models. An ordered trend across TyG quartiles was evaluated by entering quartile rank as an ordinal term. Models were adjusted for age, sex, BMI, current smoking, current alcohol consumption, occupational category, and duration of radiation-related employment. Potential non-linearity was evaluated using restricted cubic splines with knots at the 5th, 35th, 65th, and 95th percentiles of TyG and the median TyG value as the reference.

Dosimetry-substudy models included recorded cumulative occupational dose as a continuous variable and were additionally adjusted for occupational category, age, sex, BMI, current smoking, and current alcohol consumption. Total structural chromosome aberration count was the primary substudy outcome. Chromosome-type and chromatid-type counts were considered exploratory outcomes, and their two nominal *P*-values were adjusted using the Benjamini–Hochberg false discovery rate procedure. The primary total structural chromosome aberration outcome was not included in this adjustment. Inverse-probability-weighted models were used as sensitivity analyses to account for occupational-category-specific sampling. Weights were defined as the inverse of the category-specific sampling probability, normalized to have a mean of 1, and analyzed using robust sandwich variance estimators.

As a secondary analysis, heterogeneity according to occupational radiation-work status was assessed using a TyG-by-group interaction term in negative binomial regression adjusted for age, sex, BMI, current smoking, and current alcohol consumption. Healthcare controls were sampled to provide a comparison group with a similar age-by-sex distribution; no control-sampling weights were applied. Propensity score matching was conducted as a sensitivity analysis. Additional sensitivity analyses examined participant exclusions, alternative model specifications, additional adjustment for duration of radiation-related employment in the dosimetry substudy, occupational-category heterogeneity, and the association between recorded cumulative occupational dose and structural aberration count. Detailed procedures are provided in the Supplementary Methods.

TyG was standardized within the relevant analytical population: the medical radiation-worker population for primary analyses, the pooled full analytical population for the primary radiation-worker–control comparison, the matched population for the propensity score-matched analysis, and the dosimetry-substudy population for substudy analyses. Analyses were performed using R version 4.2.1 and SPSS version 29.0. All tests were two-sided. A two-sided *P*-value < 0.05 was used for the primary analysis; FDR-adjusted *q* values were reported for the exploratory subtype analyses.

## 3 Results

### 3.1 Study population and participant characteristics

Participant selection and construction of the analytical populations are shown in Figure 1. The full analytical population included 23,408 medical radiation workers and 6,542 healthcare controls. The two groups had similar age and sex distributions, consistent with the age-by-sex stratified control-sampling procedure (Table 1). The mean TyG index was 8.64 ± 0.43 among medical radiation workers and 8.58 ± 0.40 among healthcare controls. The mean structural chromosome aberration count per 100 metaphases was 0.06 ± 0.33 among medical radiation workers and 0.02 ± 0.15 among healthcare controls. At least one structural chromosome aberration was recorded in 1,036 medical radiation workers (4.4%) and 112 healthcare controls (1.7%).

**Figure 1:**
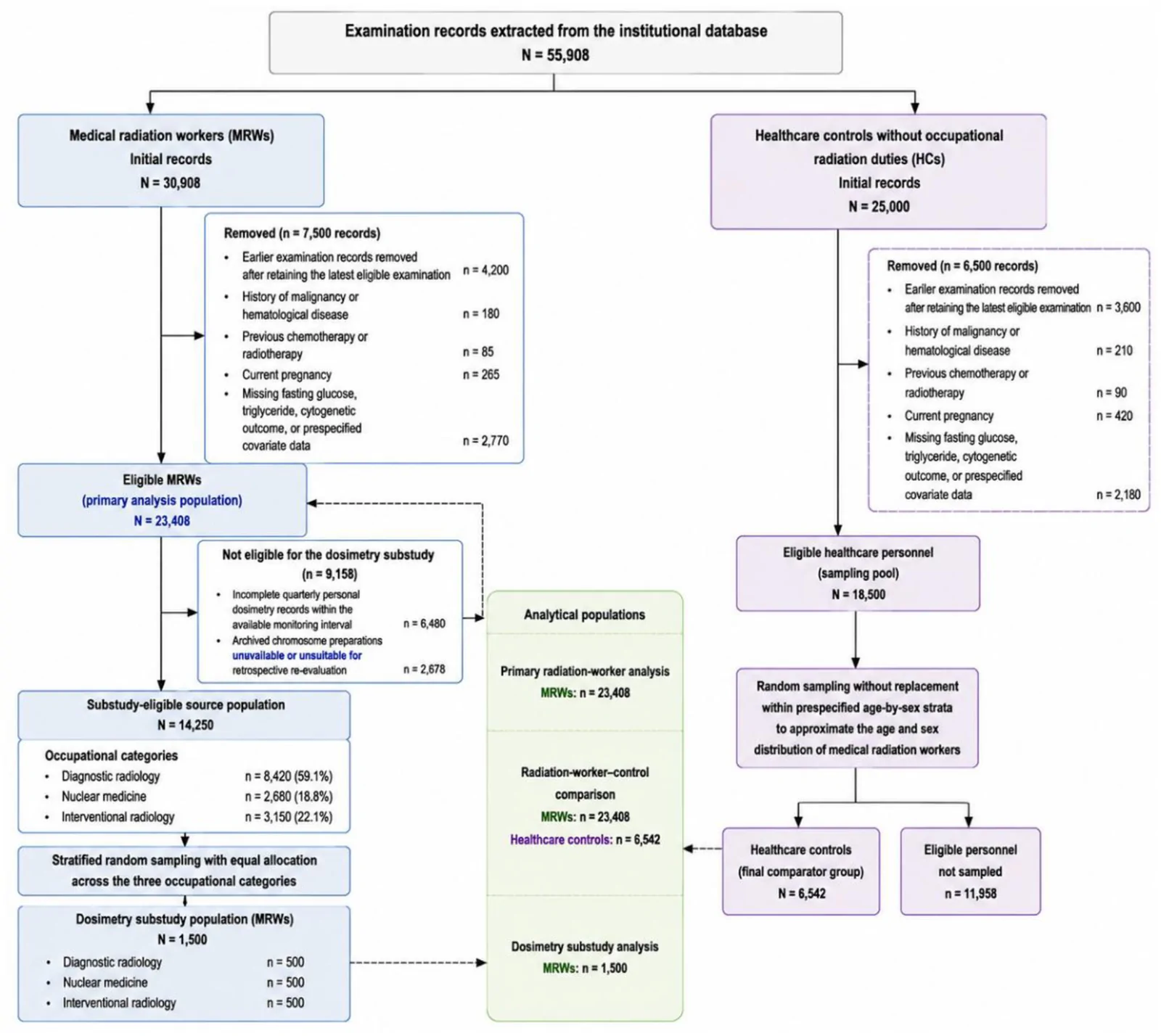
Participant selection and construction of the analytical populations. The flow diagram shows the selection of medical radiation workers and healthcare controls and the construction of the stratified dosimetry substudy.

**Table 1:** Characteristics of healthcare controls and medical radiation workers at the index examination.

Characteristic	Healthcare controls (*n* = 6,542)	Medical radiation workers (*n* = 23,408)	Absolute SMD
Age, years	41.6 ± 8.8	41.5 ± 9.0	0.011
Male sex	2,783 (42.5)	9,878 (42.2)	0.006
Current smoking	1,144 (17.5)	4,026 (17.2)	0.008
Current alcohol consumption	1,210 (18.5)	4,775 (20.4)	0.048
BMI, kg/m^2^	24.2 ± 3.0	23.8 ± 2.9	0.135
Fasting glucose, mg/dL	91.8 ± 11.2	93.5 ± 12.4	0.143
Triglycerides, mg/dL	115.4 ± 49.5	121.2 ± 54.1	0.111
TyG index	8.58 ± 0.40	8.64 ± 0.43	0.144
Structural chromosome aberrations per 100 metaphases	0.02 ± 0.15	0.06 ± 0.33	0.157
Participants with ≥1 structural chromosome aberration	112 (1.7)	1,036 (4.4)	0.156
Occupational category			
Diagnostic radiology	—	14,650 (62.6)	—
Nuclear medicine	—	4,120 (17.6)	—
Interventional radiology	—	4,638 (19.8)	—
Duration of radiation-related employment, years	—	14.9 ± 9.4	—

Healthcare controls were sampled within age-by-sex strata. Occupational variables were defined only for medical radiation workers.

Among medical radiation workers, higher TyG quartiles were characterized by older age, a higher proportion of men, higher BMI, and higher fasting glucose and triglyceride concentrations. Current smoking, current alcohol consumption, duration of radiation-related employment, and the proportion of interventional radiology workers also increased across quartiles. The mean structural chromosome aberration count increased from 0.03 ± 0.20 in Q1 to 0.10 ± 0.46 in Q4, and the proportion of participants with at least one structural chromosome aberration increased from 2.9% to 6.5% (Supplementary Table S1).

### 3.2 Association between TyG and structural chromosome aberrations among medical radiation workers

In the primary continuous analysis, each 1-SD increase in TyG was associated with a higher expected structural chromosome aberration count after adjustment for age, sex, BMI, current smoking, current alcohol consumption, occupational category, and duration of radiation-related employment (adjusted IRR, 1.15; 95% CI, 1.09–1.22; Table 2).

**Table 2:** Association between the TyG index and structural chromosome aberration outcomes among medical radiation workers (*n* = 23,408).

TyG parameterization	Structural chromosome aberration count, adjusted IRR (95% CI)	Presence of ≥1 structural chromosome aberration, adjusted OR (95% CI)
Per 1-SD increase	1.15 (1.09–1.22)	1.25 (1.16–1.35)
Q1 (< 8.33)	1.00 (Reference)	1.00 (Reference)
Q2 (8.33– < 8.63)	1.10 (0.91–1.33)	1.15 (0.92–1.43)
Q3 (8.63– < 8.92)	1.27 (1.05–1.53)	1.38 (1.12–1.70)
Q4 (≥8.92)	1.52 (1.27–1.83)	1.85 (1.50–2.28)
*P* for trend	< 0.001	< 0.001

Models were adjusted for age, sex, BMI, current smoking, current alcohol consumption, occupational category, and duration of radiation-related employment. Continuous TyG and TyG quartiles were modeled separately; 1 SD of TyG was 0.43.

The quartile analysis showed a graded association. Compared with Q1, the adjusted IRRs for structural chromosome aberration count were 1.10 (95% CI, 0.91–1.33) for Q2, 1.27 (95% CI, 1.05–1.53) for Q3, and 1.52 (95% CI, 1.27–1.83) for Q4 (*P* for trend < 0.001).

Results for the complementary binary outcome were directionally consistent. Each 1-SD increase in TyG was associated with higher odds of having at least one structural chromosome aberration (adjusted OR, 1.25; 95% CI, 1.16–1.35). Compared with Q1, the adjusted ORs were 1.15 (95% CI, 0.92–1.43) for Q2, 1.38 (95% CI, 1.12–1.70) for Q3, and 1.85 (95% CI, 1.50–2.28) for Q4 (*P* for trend < 0.001).

Restricted cubic spline analysis indicated evidence of non-linearity (*P* for non-linearity = 0.013), with the estimated association becoming steeper at higher TyG values (Figure 2).

**Figure 2:**
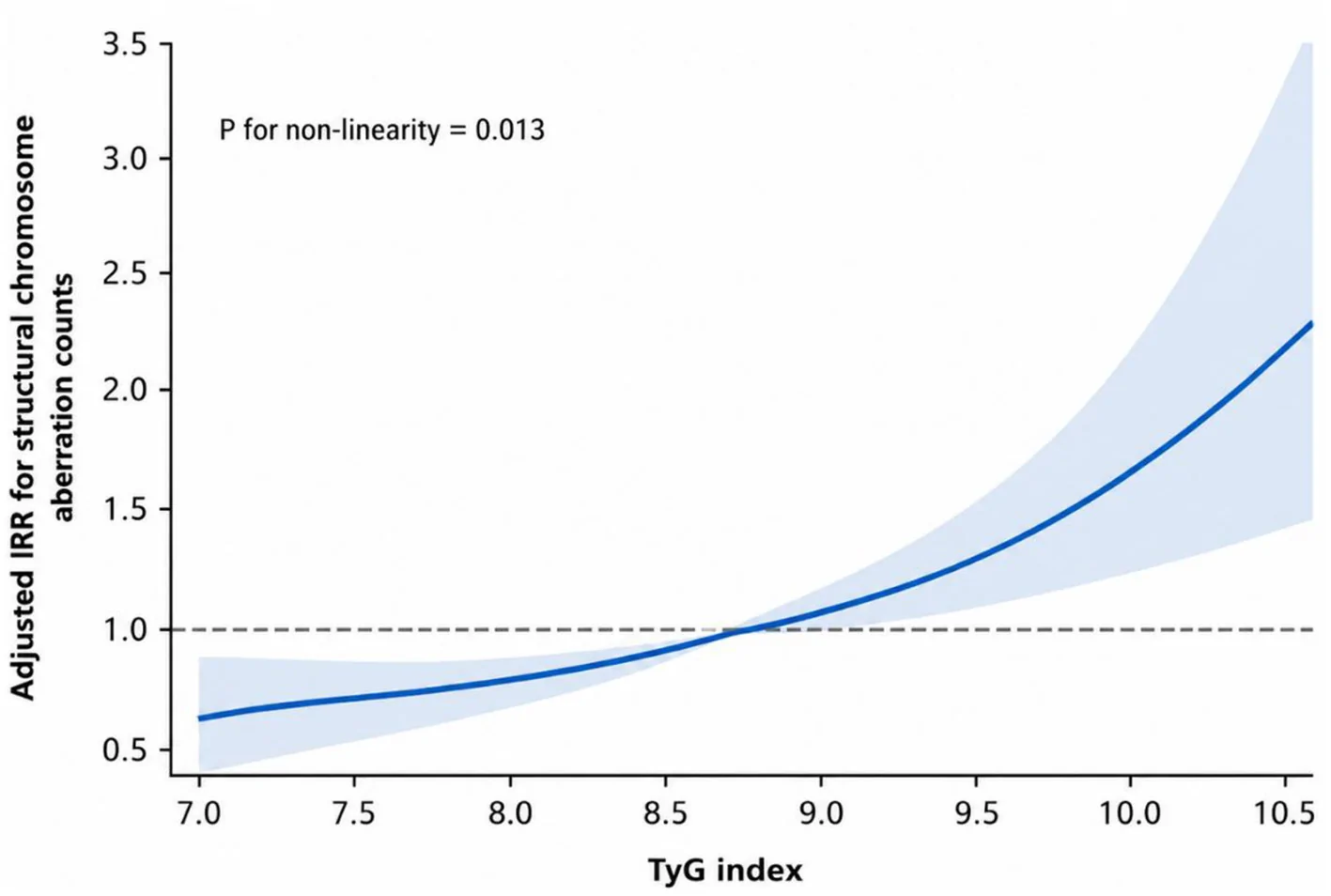
Restricted cubic spline association between the TyG index and structural chromosome aberration count among medical radiation workers. The solid line represents the adjusted IRR, and the shaded area represents the 95% CI. The median TyG value was used as the reference.

### 3.3 Stratified dosimetry substudy

The dosimetry substudy included 1,500 medical radiation workers, with 500 participants in each occupational category. Participant characteristics and category-specific dose distributions are presented in Supplementary Table S2. The median recorded cumulative occupational dose was 28.5 mSv (IQR, 15.2–54.3 mSv), with category-specific medians of 12.4 mSv (IQR, 8.1–19.5 mSv) for diagnostic radiology, 32.1 mSv (IQR, 22.4–45.8 mSv) for nuclear medicine, and 61.3 mSv (IQR, 45.2–82.4 mSv) for interventional radiology. Category-specific sampling probabilities and normalized inverse-probability weights are presented in Supplementary Table S3.

Blinded re-evaluation identified 194 structural chromosome aberration events among 132 participants with at least one aberration (8.8%), comprising 86 chromosome-type and 108 chromatid-type events. Because the full-population outcome was obtained from routine aggregate records whereas the substudy outcome was based on blinded re-evaluation of archived preparations, absolute aberration frequencies were not directly compared between the two analytical settings. Representative structural chromosome aberrations identified during the substudy re-evaluation are shown in Figure 3.

**Figure 3:**
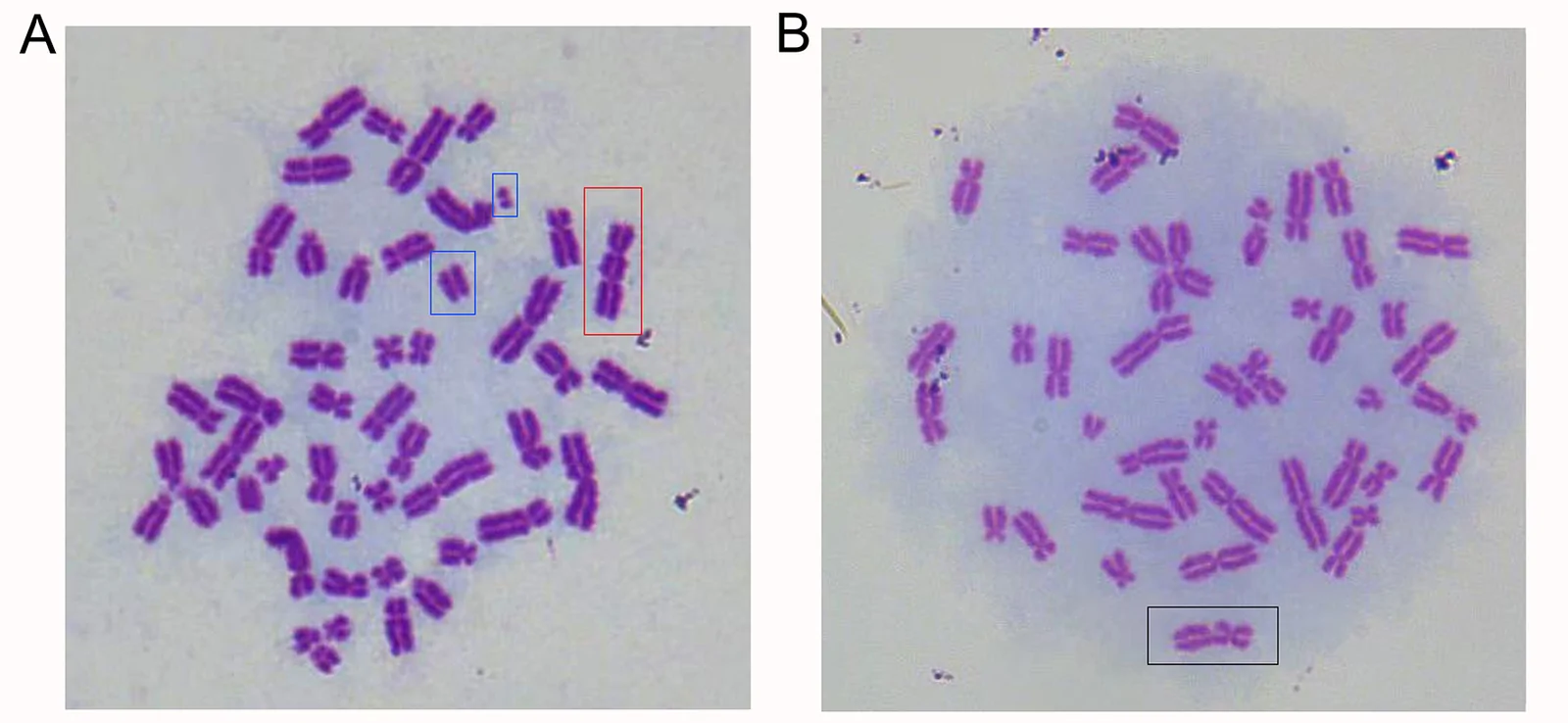
Representative structural chromosome aberrations identified during blinded cytogenetic re-evaluation in the dosimetry substudy. Giemsa-stained metaphase spreads are shown. **(A)** A representative metaphase spread showing one dicentric chromosome (red box) and two acentric fragments (blue boxes). **(B)** A representative metaphase spread showing a chromatid break (black box).

In multivariable negative binomial regression, each 1-SD increase in TyG was associated with a higher total structural chromosome aberration count after adjustment for recorded cumulative occupational dose, occupational category, age, sex, BMI, current smoking, and current alcohol consumption (adjusted IRR, 1.27; 95% CI, 1.14–1.42; Table 3). Associations were also observed for the exploratory chromosome-type (86 events; adjusted IRR, 1.28; 95% CI, 1.12–1.47) and chromatid-type outcomes (108 events; adjusted IRR, 1.22; 95% CI, 1.07–1.38), and both remained statistically significant after FDR correction (both *q* < 0.01). Inverse-probability-weighted estimates were similar for the total, chromosome-type, and chromatid-type outcomes (Table 3).

**Table 3:** Association between the TyG index and structural chromosome aberration counts in the stratified dosimetry substudy (*n* = 1,500).

Aberration outcome	Total aberration events, *n*	Participants with ≥1 event, *n* (%)	Adjusted IRR per 1-SD increase in TyG (95% CI)	Nominal *P* value	FDR-adjusted *q* value	IPW-adjusted IRR (95% CI)
Total structural chromosome aberrations	194	132 (8.8)	1.27 (1.14–1.42)	< 0.001	—	1.25 (1.11–1.41)
Chromosome-type aberrations	86	64 (4.3)	1.28 (1.12–1.47)	< 0.001	< 0.01	1.26 (1.08–1.46)
Chromatid-type aberrations	108	82 (5.5)	1.22 (1.07–1.38)	0.002	< 0.01	1.20 (1.04–1.39)

Models were adjusted for recorded cumulative occupational dose, occupational category, age, sex, BMI, current smoking, and current alcohol consumption. Nominal *P*-values and FDR-adjusted *q* values correspond to the unweighted adjusted models. FDR adjustment was applied only to the two subtype analyses. IPW estimates used normalized inverse occupational-category-specific sampling weights and robust sandwich variance estimators. One SD of TyG was 0.45. Participants could contribute to both subtype categories.

Recorded cumulative occupational dose was positively associated with total structural chromosome aberration count. The adjusted IRR was 1.11 (95% CI, 1.05–1.18) per 10-mSv increase before inclusion of TyG and 1.10 (95% CI, 1.04–1.17) after additional adjustment for TyG. The corresponding inverse-probability-weighted estimate was 1.10 (95% CI, 1.03–1.18; Supplementary Table S4).

### 3.4 Secondary analysis according to occupational radiation-work status

In the full-population interaction model, in which TyG was standardized within the pooled analytical population, the adjusted IRR per 1-SD increase in TyG was 1.15 (95% CI, 1.09–1.22) among medical radiation workers and 1.02 (95% CI, 0.88–1.18) among healthcare controls. The ratio of the TyG-associated IRRs was 1.13 (95% CI, 1.03–1.24; *P* for interaction = 0.012; Supplementary Table S5).

Propensity score matching yielded 6,214 matched pairs, with all post-matching absolute SMDs < 0.10 (Supplementary Table S6). After TyG was re-standardized within the matched population, the adjusted IRRs per 1-SD increase were 1.16 (95% CI, 1.09–1.24) among medical radiation workers and 1.04 (95% CI, 0.90–1.20) among healthcare controls. The corresponding ratio of IRRs was 1.12 (95% CI, 1.02–1.22; *P* for interaction = 0.021; Supplementary Table S5).

### 3.5 Sensitivity analyses

The primary association was similar across sensitivity analyses (Supplementary Table S7). After excluding 1,124 participants with self-reported diabetes or current lipid-lowering therapy, the adjusted IRR was 1.13 (95% CI, 1.07–1.21) per 1-SD increase in TyG and 1.45 (95% CI, 1.21–1.74) for Q4 vs. Q1. Of the excluded participants, 57.3% were in Q4, and the prevalence of exclusion increased across TyG quartiles (Supplementary Table S8). Estimates were also similar when BMI was modeled categorically and when zero-inflated negative binomial regression was used.

In the dosimetry substudy, additional adjustment for duration of radiation-related employment produced an adjusted IRR of 1.26 (95% CI, 1.13–1.41) per 1-SD increase in TyG (Supplementary Table S9). Category-specific estimates were 1.21 (95% CI, 0.95–1.54) for diagnostic radiology, 1.25 (95% CI, 1.05–1.49) for nuclear medicine, and 1.31 (95% CI, 1.11–1.55) for interventional radiology, with no evidence of heterogeneity across occupational categories (*P* for interaction = 0.628; Supplementary Table S10).

## 4 Discussion

In this retrospective cross-sectional study, a higher TyG index was associated with a greater structural chromosome aberration burden among medical radiation workers. The association was evident in continuous, quartile-based, and spline analyses and remained after adjustment for recorded cumulative occupational dose in the stratified dosimetry substudy. Inverse-probability-weighted estimates were similar. These findings identify TyG as a correlate of cytogenetic variation but do not establish temporality, causality, or individual radiosensitivity.

Most previous epidemiological studies of TyG have focused on cardiovascular and other cardiometabolic outcomes (22–25), whereas evidence regarding cytogenetic endpoints remains limited. The present findings are biologically plausible but should be interpreted as hypothesis-generating. Insulin resistance and related metabolic disturbances have been associated with oxidative stress and chronic low-grade inflammation (13–15), while nutrient- and insulin-related signaling pathways are involved in cellular responses to DNA damage and repair processes (16–18). These oxidative and inflammatory processes provide biological context for investigating the observed association between metabolic status and structural chromosome aberration burden. However, oxidative stress, inflammatory activity, insulin resistance assessed by reference methods, and DNA repair capacity were not evaluated in this study. Therefore, the observed association cannot be attributed to any specific biological pathway, and TyG should be interpreted as a readily available metabolic marker associated with cytogenetic variation rather than a direct indicator of DNA damage or repair capacity. Similarly, the non-linear association observed in spline analysis does not establish a biological threshold and should not be interpreted as supporting a TyG cutoff for occupational screening.

The dosimetry substudy provides important context for interpreting the primary findings. Recorded cumulative occupational dose was positively associated with structural chromosome aberration count, consistent with the established role of ionizing radiation in inducing chromosome damage and supporting the continued primacy of physical dosimetry in occupational radiation protection. The association between TyG and chromosome aberration count remained after adjustment for recorded cumulative occupational dose, whereas additional adjustment for TyG only slightly attenuated the dose-associated estimate. This pattern suggests that recorded cumulative occupational dose and TyG may provide partly distinct statistical information regarding variation in chromosome aberration burden. However, it does not demonstrate that TyG has a radiation-independent causal effect, because recorded cumulative occupational dose represents an incomplete measure of biologically relevant radiation exposure. The similarity between weighted and unweighted estimates indicates that accounting for the unequal sampling fractions across occupational categories did not materially alter the TyG-associated estimate. Nevertheless, estimates from the substudy should not be directly compared with those from the full population because the full-population outcome was obtained from routine aggregate records, whereas the substudy outcome was based on blinded re-evaluation of archived preparations.

The interaction estimate was modest, and the control-specific association was imprecisely estimated because relatively few healthcare controls had nonzero aberration counts. This secondary finding therefore requires confirmation in studies with more informative cytogenetic outcome distributions in comparison populations. Occupational group is a broad indicator that may reflect differences in exposure patterns, work practices, lifestyle factors, and other occupational characteristics. Similarly, the associations observed for chromosome-type and chromatid-type aberrations were based on relatively few events and should be considered exploratory. Conventional Giemsa-based scoring also does not comprehensively detect stable aberrations such as reciprocal translocations or inversions, and future studies incorporating molecular cytogenetic approaches are needed.

The findings may have relevance for the interpretation of cytogenetic surveillance data but do not currently support a change in occupational radiation-protection practice. TyG is readily derived from routine fasting measurements and may represent one metabolic characteristic associated with between-person variation in chromosome aberration counts. However, the absolute aberration counts were low, the clinical and long-term prognostic significance of the observed differences is uncertain, and no threshold for individual decision-making was evaluated. TyG should therefore not be used as a biomarker of radiation dose, an indicator of individual radiosensitivity, a basis for modifying occupational dose limits, or a criterion for fitness for radiation work. Its potential value lies instead in motivating prospective research on how metabolic health may relate to cytogenetic outcomes in occupationally exposed populations.

Several limitations should be considered. First, the cross-sectional design and concurrent assessment of TyG and chromosome aberrations prevent determination of temporal relationships, and a single TyG measurement may not reflect long-term metabolic status. Second, residual confounding remains possible because information on lifestyle factors, inflammatory status, medication use, chemical co-exposures, and non-occupational radiation exposure was incomplete. Specifically, diet, physical activity, and inflammatory biomarkers were not systematically available, and no sufficiently complete validated proxy measures were identified. Depending on their relationships with TyG and chromosome aberration burden, these unmeasured factors could have inflated or attenuated the observed association. Third, recorded cumulative occupational dose did not necessarily capture the full history or biological relevance of radiation exposure. Because dosimeter placement and protective-equipment use were not comprehensively available, exposure measurement error may differ across occupational categories, particularly in interventional radiology, and may contribute to residual exposure misclassification. Finally, the single-center design and eligibility requirements of the dosimetry substudy may limit generalizability. Prospective multicenter studies incorporating repeated metabolic assessments, comprehensive dosimetry, and mechanistic biomarkers are needed to establish reproducibility and occupational-health relevance.

## 5 Conclusion

Higher TyG was associated with a greater structural chromosome aberration count among medical radiation workers, including in the stratified dosimetry substudy after adjustment for recorded cumulative occupational dose. These findings identify TyG as a metabolic correlate of cytogenetic variation but do not establish temporality, causality, or individual radiosensitivity. Prospective studies with repeated metabolic and cytogenetic assessments and more comprehensive occupational dosimetry are needed to determine the reproducibility and occupational-health relevance of this association.

## Data Availability

The datasets analyzed in this study are not publicly available because of institutional privacy restrictions. De-identified data may be made available by the corresponding author upon reasonable request and subject to institutional approval.
