# Supplementary material for: Triglyceride–glucose index and structural chromosome aberrations in medical radiation workers: a cross-sectional study with a stratified dosimetry substudy
Source: Front Public Health. 2026 Sep 14;14:1908697. doi: 10.3389/fpubh.2026.1908697 (PMC13616680; doi:10.3389/fpubh.2026.1908697)
Supplement: Supplementary file 1 [file Supplementary_File_1.docx]

**Supplementary Methods**

**Control sampling**

Healthcare personnel without documented occupational radiation duties were identified from the standardized institutional health-examination database for the same study period. Of 25,000 initial examination records, 3,600 earlier examinations from individuals with multiple records were removed, with only the latest eligible examination retained. A further 210 records were excluded because of malignancy or hematological disease, 90 because of previous chemotherapy or radiotherapy, 420 because of current pregnancy, and 2,180 because of missing fasting glucose, triglyceride, cytogenetic outcome, or prespecified covariate data. This process yielded 18,500 eligible healthcare personnel in the control sampling pool.

Eligible personnel were stratified by sex and prespecified age categories. The number of controls selected from each age-by-sex stratum was determined proportionally according to the distribution of medical radiation workers in the corresponding strata. Within each stratum, controls were selected by computer-generated simple random sampling without replacement, yielding 6,542 healthcare controls; the remaining 11,958 eligible personnel were not sampled. This procedure represented group-level stratified sampling rather than individual matching.

**Medical radiation-worker selection**

Medical radiation workers were identified from the occupational health-surveillance database. Of 30,908 initial examination records, 4,200 earlier examination records from individuals with multiple eligible examinations were removed after retention of the latest eligible examination. A further 180 records were excluded because of malignancy or hematological disease, 85 because of previous chemotherapy or radiotherapy, 265 because of current pregnancy, and 2,770 because of missing fasting glucose, triglyceride, cytogenetic outcome, or prespecified covariate data. This process yielded 23,408 eligible medical radiation workers for the primary analysis.

**Cytogenetic culture and scoring procedures**

Peripheral blood lymphocytes from medical radiation workers and healthcare controls were cultured and processed through the institutional cytogenetics laboratory according to standard operating procedures based on GBZ/T 248-2014. Briefly, 0.3–0.5 mL of sodium-heparinized peripheral blood was added to approximately 4.5 mL of RPMI-1640 medium supplemented with 15%–20% fetal bovine serum and phytohemagglutinin at a final concentration of 10–20 μg/mL. Cultures were incubated at 37°C for 48 h, and metaphase arrest was induced during the final 2–3 h using colcemid at a final concentration of 0.05–0.10 μg/mL.

Cells were harvested after hypotonic treatment with prewarmed 0.075 mol/L KCl at 37°C for 20–30 min and fixed three times in freshly prepared methanol–acetic acid solution (3:1, v/v). Air-dried slides were stained with 5%–10% Giemsa solution prepared in phosphate buffer (pH 6.8) for 10–15 min. The reported ranges reflect minor procedural variations across contemporaneous versions of the laboratory standard operating procedure used during the study period.

For each participant, 100 well-spread metaphases with adequate chromosome morphology were evaluated. Metaphases with approximately diploid chromosome numbers (46 ± 1), adequate staining, and no substantial chromosome overlap or apparent chromosome loss due to poor spreading were considered eligible for scoring. Chromatid gaps and isolated acentric fragments were excluded. An acentric fragment accompanying a dicentric chromosome or centric ring was recorded as part of the corresponding event and was not counted as an additional independent event.

For the full analytical population, aggregate structural chromosome aberration counts were obtained from the standardized institutional cytogenetic database. Routine records retained the aggregate structural aberration count per 100 metaphases based on the same event definitions but did not consistently preserve sufficiently detailed subtype labels for retrospective subtype analysis. Consequently, full-population analyses were restricted to the aggregate count and the presence of at least one recorded structural aberration.

For the dosimetry substudy, archived preparations were independently re-evaluated by two experienced cytogeneticists who were blinded to participants’ TyG values, occupational categories, and recorded cumulative occupational doses. Exactly 100 eligible metaphases were evaluated for each participant. Chromosome-type aberrations included dicentric chromosomes and centric rings. Chromatid-type aberrations included chromatid breaks and chromatid exchanges, including radial configurations. Disagreements regarding the presence or classification of an aberration were resolved by joint review, and the consensus classification was retained. Re-evaluated counts were used only in the dosimetry-substudy analyses and were not used to recalibrate the routine aggregate records for the full analytical population.

Routine cytogenetic scoring and biochemical testing were conducted through separate laboratory workflows, and TyG values were not used in cytogenetic interpretation. Conventional Giemsa-based assessment primarily identified unstable chromosome-type aberrations and morphologically recognizable chromatid-type aberrations. Stable chromosome-type aberrations, including reciprocal translocations and inversions, could not be comprehensively ascertained and were not included in the subtype analyses.

**Dosimetry procedures and exposure measurement**

Occupational external radiation exposure was monitored using personal thermoluminescent or optically stimulated luminescence dosimeters supplied and processed by certified occupational radiation monitoring agencies operating under national quality-assurance procedures. Dosimeters were exchanged quarterly; therefore, each agency-reported value represented the dose accumulated over a three-month monitoring period.

For each participant, the dosimetry observation window extended from the first available valid quarterly record to the last completed monitoring quarter before cytogenetic sampling. Recorded cumulative occupational dose was calculated as the sum of all valid agency-reported quarterly values within this participant-specific interval. Workers with one or more missing quarterly monitoring records between the first and last included records were not eligible for the substudy. Thus, record completeness referred to an uninterrupted sequence within the available observation window and did not necessarily represent complete coverage of the participant’s entire radiation-related employment history.

Quarterly dose values were analyzed as reported by the monitoring agencies. The study investigators did not retrospectively impute or substitute alternative values for low or below-threshold readings. Because detector-specific reporting thresholds and historical below-threshold coding conventions were not consistently documented in a harmonized form across monitoring systems and periods, a single uniform reporting threshold could not be verified retrospectively. Recorded cumulative occupational dose was therefore interpreted as the sum of agency-reported values over the available monitoring interval rather than as a reconstruction of lifetime occupational exposure.

Recorded cumulative occupational dose was entered as a continuous variable in the dosimetry-substudy regression models. Associations between recorded cumulative occupational dose and structural aberration count were reported per 10-mSv increase.

The available records represented routine personal external dose monitoring. Organ-specific, internal, and task-specific exposures, including lens and extremity doses, fluoroscopy time, dosimeter placement, protective-equipment use, worker position, and procedure-specific dose rates, were not comprehensively available.

**Propensity score matching**

Propensity scores for occupational radiation-work status were estimated using logistic regression including age, sex, BMI, current smoking, and current alcohol consumption. Medical radiation workers and healthcare controls were matched 1:1 using nearest-neighbor matching without replacement and a caliper width of 0.2 SD of the logit of the propensity score. Covariate balance was evaluated using absolute SMDs, with values <0.10 indicating adequate balance.

The matched-cohort negative binomial model included continuous TyG, occupational group, their multiplicative interaction term, and the prespecified covariates. Cluster-robust sandwich variance estimators based on matched-pair identifiers were used to account for within-pair dependence.

**Inverse-probability weighting**

Occupational-category-specific sampling probabilities were calculated by dividing the number of selected participants by the number of eligible workers within each category. Raw inverse-probability weights were defined as the reciprocal of the corresponding sampling probabilities and were normalized to a mean of 1 among substudy participants. No weight trimming or truncation was applied.

Inverse-probability-weighted negative binomial models retained the same covariates as the corresponding unweighted dosimetry models and used robust sandwich variance estimators. Weighting standardized the occupational-category distribution to that of the 14,250 substudy-eligible workers. It did not account for eligibility associated with uninterrupted dosimetry records or the availability of archived chromosome preparations.

**Sensitivity and secondary analyses**

Primary radiation-worker count models were repeated after excluding participants with self-reported diabetes or current lipid-lowering medication use. TyG quartile boundaries from the original radiation-worker population were retained after these exclusions. An additional analysis replaced continuous BMI with Chinese clinical BMI categories of <24.0, 24.0–27.9, and ≥28.0 kg/m².

A zero-inflated negative binomial model was fitted to assess robustness to excess zeros in the structural aberration count distribution. The count component included TyG and the same covariates as the primary negative binomial model, whereas the zero-inflation component included an intercept only. Continuous and quartile-based TyG terms were entered into separate models. Exponentiated coefficients from the count component were reported as IRRs.

In the dosimetry substudy, the primary model did not include duration of radiation-related employment because age, employment duration, and recorded cumulative occupational dose represented related temporal measures. Duration of radiation-related employment was added in a sensitivity model. Occupational-category-specific TyG associations were estimated using models adjusted for recorded cumulative occupational dose, age, sex, BMI, current smoking, and current alcohol consumption. Heterogeneity across the three occupational categories was evaluated using a joint Wald test of the TyG-by-occupational-category interaction terms.

The association between recorded cumulative occupational dose and total structural chromosome aberration count was evaluated using negative binomial regression, with dose expressed per 10-mSv increase. The first model adjusted for occupational category, age, sex, BMI, current smoking, and current alcohol consumption; continuous TyG was added in the second model. A corresponding inverse-probability-weighted model used normalized occupational-category-specific weights and robust sandwich variance estimators.

Chromosome-type and chromatid-type aberration counts were treated as exploratory outcomes and analyzed using the same covariates as the primary dosimetry model. Their two nominal P values constituted a separate multiple-testing family and were adjusted using the Benjamini–Hochberg false discovery rate procedure. The primary total structural chromosome aberration outcome was not included in this adjustment.

**Statistical implementation**

Analyses were based on participants with complete data for the outcome, TyG index, and prespecified covariates; missing values were not imputed. For continuous variables, absolute SMDs were calculated as the absolute difference in means divided by the pooled SD. For binary variables, the absolute difference in proportions was divided by the pooled binomial SD.

TyG was standardized by subtracting the relevant analytical-population mean and dividing by its SD. Standardization was based on the medical radiation-worker population for the primary analyses, the pooled full analytical population for the primary radiation-worker–control interaction analysis, the propensity score-matched population for the matched analysis, and the overall dosimetry-substudy population for the substudy analyses.

The variance of the structural aberration count exceeded its mean, indicating overdispersion. Accordingly, the count was analyzed using negative binomial regression. Exponentiated regression coefficients were reported as IRRs by model convention and represent ratios of expected structural chromosome aberration counts per 100 evaluated metaphases rather than incidence rates over person-time. No offset term was included because exactly 100 metaphases were evaluated for each participant. TyG quartiles and continuous TyG were entered into separate models, and an ordered trend across quartiles was evaluated by entering quartile rank as an ordinal variable.

Potential non-linearity was evaluated using restricted cubic splines with knots at the 5th, 35th, 65th, and 95th percentiles of the TyG distribution and the median as the reference. The P value for non-linearity was obtained by jointly testing the nonlinear spline terms.

Effect heterogeneity according to occupational radiation-work status was evaluated using a model containing continuous TyG, occupational group, and their multiplicative interaction term. Healthcare controls were the reference group. The exponentiated interaction coefficient represented the ratio of the TyG-associated IRR among medical radiation workers to that among healthcare controls.

All statistical tests were two-sided. Analyses were conducted using R version 4.2.1 and SPSS version 29.0.
