# Supplementary material for: Triglyceride–glucose index and structural chromosome aberrations in medical radiation workers: a cross-sectional study with a stratified dosimetry substudy
Source: Front Public Health. 2026 Sep 14;14:1908697. doi: 10.3389/fpubh.2026.1908697 (PMC13616680; doi:10.3389/fpubh.2026.1908697)
Supplement: Supplementary file 2 [file Table_1.docx]

**Supplementary Tables**

**Supplementary Table S1. Characteristics of medical radiation workers according to TyG quartile (*n* = 23,408)**

| **Characteristic** | **Q1 (< 8.33; *n* = 5,852)** | **Q2 (8.33 to <8.63; *n* = 5,852)** | **Q3 (8.63 to <8.92; *n* = 5,852)** | **Q4 (≥8.92; *n* = 5,852)** |
| --- | --- | --- | --- | --- |
| Age, years | 38.7 ± 8.7 | 40.8 ± 8.9 | 42.6 ± 9.1 | 43.7 ± 9.4 |
| Male sex | 2,050 (35.0) | 2,270 (38.8) | 2,508 (42.9) | 3,050 (52.1) |
| BMI, kg/m^2^ | 22.2 ± 2.3 | 23.3 ± 2.5 | 24.2 ± 2.8 | 25.5 ± 2.9 |
| Current smoking | 830 (14.2) | 910 (15.5) | 1,060 (18.1) | 1,226 (21.0) |
| Current alcohol consumption | 970 (16.6) | 1,100 (18.8) | 1,255 (21.4) | 1,450 (24.8) |
| Duration of radiation-related employment, years | 13.1 ± 8.9 | 14.2 ± 9.1 | 15.6 ± 9.4 | 16.7 ± 9.9 |
| Occupational category |  |  |  |  |
| Diagnostic radiology | 3,804 (65.0) | 3,745 (64.0) | 3,629 (62.0) | 3,472 (59.3) |
| Nuclear medicine | 1,030 (17.6) | 1,040 (17.8) | 1,030 (17.6) | 1,020 (17.4) |
| Interventional radiology | 1,018 (17.4) | 1,067 (18.2) | 1,193 (20.4) | 1,360 (23.2) |
| Fasting glucose, mg/dL | 88.1 ± 8.8 | 91.4 ± 10.2 | 94.9 ± 11.5 | 99.6 ± 15.1 |
| Triglycerides, mg/dL | 78.2 ± 18.0 | 104.0 ± 25.5 | 130.6 ± 35.0 | 172.0 ± 68.5 |
| TyG index | **8.08 ± 0.14** | **8.49 ± 0.12** | **8.82 ± 0.12** | **9.17 ± 0.21** |
| Structural chromosome aberrations per 100 metaphases | **0.03 ± 0.20** | **0.04 ± 0.26** | **0.07 ± 0.34** | **0.10 ± 0.46** |
| Participants with ≥1 structural chromosome aberration | 170 (2.9) | 214 (3.7) | 269 (4.6) | 383 (6.5) |

Note: TyG quartiles were defined in the medical radiation-worker analytical population.

**Supplementary Table S2. Characteristics and recorded cumulative occupational dose of the full medical radiation-worker population and the stratified dosimetry substudy**

| **Characteristic** | **Full radiation-worker population (n = 23,408)** | **Stratified dosimetry substudy (n = 1,500)** |
| --- | --- | --- |
| Age, years | 41.5 ± 9.0 | 42.1 ± 8.8 |
| Male sex | 9,878 (42.2) | 705 (47.0) |
| BMI, kg/m² | 23.8 ± 2.9 | 24.1 ± 3.0 |
| TyG index | 8.64 ± 0.43 | 8.68 ± 0.45 |
| Duration of radiation-related employment, years | 14.9 ± 9.4 | 15.4 ± 9.2 |
| Occupational category |  |  |
| Diagnostic radiology | 14,650 (62.6) | 500 (33.3) |
| Nuclear medicine | 4,120 (17.6) | 500 (33.3) |
| Interventional radiology | 4,638 (19.8) | 500 (33.3) |
| Recorded cumulative occupational dose, mSv | — | 28.5 (15.2–54.3) |
| Recorded cumulative occupational dose by category, mSv |  |  |
| Diagnostic radiology | — | 12.4 (8.1–19.5) |
| Nuclear medicine | — | 32.1 (22.4–45.8) |
| Interventional radiology | — | 61.3 (45.2–82.4) |

Note: The substudy used equal allocation across the three occupational categories; therefore, its unweighted occupational-category distribution differed from that of the full medical radiation-worker population.

**Supplementary Table S3. Sampling probabilities and inverse-probability weights in the stratified dosimetry substudy**

| **Occupational category** | **Eligible source population, n** | **Selected participants, n** | **Sampling probability** | **Raw inverse-probability weight** | **Normalized weight** |
| --- | --- | --- | --- | --- | --- |
| Diagnostic radiology | 8,420 | 500 | 0.05938 | 16.840 | 1.773 |
| Nuclear medicine | 2,680 | 500 | 0.18657 | 5.360 | 0.564 |
| Interventional radiology | 3,150 | 500 | 0.15873 | 6.300 | 0.663 |
| Overall | 14,250 | 1,500 | — | Mean: 9.500 | Mean: 1.000 |

Note: Weights standardized the occupational-category distribution to that of the 14,250 substudy-eligible workers. They did not account for eligibility related to dosimetry-record completeness or archived-preparation availability.

**Supplementary Table S4. Association between recorded cumulative occupational dose and total structural chromosome aberration count in the stratified dosimetry substudy**

| **Model** | **Dose scale** | **Adjusted IRR (95% CI)** | ***P* value** |
| --- | --- | --- | --- |
| Base model | Per 10 mSv | 1.11 (1.05–1.18) | <0.001 |
| Additionally adjusted for TyG | Per 10 mSv | 1.10 (1.04–1.17) | 0.001 |
| IPW model additionally adjusted for TyG | Per 10 mSv | 1.10 (1.03–1.18) | 0.006 |

Note: The base model was adjusted for occupational category, age, sex, BMI, current smoking, and current alcohol consumption. TyG was included in the second and IPW models. The IRR represents the ratio of expected structural chromosome aberration counts per 10-mSv increase in recorded cumulative occupational dose. The IPW model accounted for occupational-category-specific sampling.

**Supplementary Table S5. Parameter estimates from interaction models of TyG and occupational radiation-work status**

**Part A: Full analytical population (*n* = 29,950)**

| **Parameter** | **Estimate (β)** | **SE** | **Wald χ^2^** | ***P* value** | **IRR(95% CI)** |
| --- | --- | --- | --- | --- | --- |
| Intercept | -4.685 | 0.412 | 129.35 | <0.001 | — |
| TyG, per 1-SD increase | 0.0198 | 0.0748 | 0.07 | 0.791 | 1.02 (0.88–1.18) |
| Medical radiation-worker status (medical radiation worker = 1) | 0.963 | 0.126 | 58.46 | <0.001 | 2.62 (2.05–3.35) |
| TyG × occupational-group interaction | 0.120 | 0.0478 | 6.30 | 0.012 | 1.13 (1.03–1.24) |
| Age, per 10-year increase | 0.067 | 0.030 | 4.98 | 0.025 | 1.07 (1.01–1.14) |
| Male sex | 0.039 | 0.058 | 0.45 | 0.501 | 1.04 (0.93–1.17) |
| BMI, per 1 kg/m² increase | 0.019 | 0.009 | 4.46 | 0.035 | 1.02 (1.00–1.04) |
| Current smoking | 0.205 | 0.056 | 13.40 | <0.001 | 1.23 (1.10–1.37) |
| Current alcohol consumption | 0.048 | 0.055 | 0.76 | 0.383 | 1.05 (0.94–1.17) |

**Part B: Propensity score-matched population (*n*=12,428)**

| **Parameter** | **Estimate (β)** | **SE** | **Wald χ^2^** | ***P* value** | **IRR (95% CI)** |
| --- | --- | --- | --- | --- | --- |
| Intercept | -4.512 | 0.534 | 71.39 | <0.001 | — |
| TyG, per 1-SD increase | 0.0392 | 0.0734 | 0.28 | 0.596 | 1.04 (0.90–1.20) |
| Medical radiation-worker status (medical radiation worker = 1) | 0.885 | 0.132 | 44.95 | <0.001 | 2.42 (1.87–3.13) |
| TyG × occupational-group interaction | 0.109 | 0.0473 | 5.31 | 0.021 | 1.12 (1.02–1.22) |
| Age, per 10-year increase | 0.061 | 0.042 | 2.11 | 0.146 | 1.06 (0.98–1.15) |
| Male sex | 0.045 | 0.075 | 0.36 | 0.548 | 1.05 (0.91–1.21) |
| BMI, per 1 kg/m² increase | 0.018 | 0.012 | 2.25 | 0.133 | 1.02 (0.99–1.04) |
| Current smoking | 0.184 | 0.072 | 6.53 | 0.011 | 1.20 (1.04–1.38) |
| Current alcohol consumption | 0.065 | 0.071 | 0.83 | 0.362 | 1.07 (0.93–1.23) |

Note: Healthcare controls were the reference group. TyG was standardized within the full pooled analytical population in Part A and within the propensity score-matched population in Part B. The exponentiated interaction coefficient represents the ratio of the TyG-associated IRR among medical radiation workers to that among healthcare controls. The matched analysis used cluster-robust variance estimators based on matched-pair identifiers.

**Supplementary Table S6. Covariate balance in the propensity score-matched population**

| **Characteristic** | **Healthcare controls (n = 6,214)** | **Medical radiation workers (n = 6,214)** | **Absolute SMD** |
| --- | --- | --- | --- |
| Age, years | 41.5 ± 8.8 | 41.5 ± 8.9 | 0.003 |
| Male sex | 2,632 (42.4) | 2,648 (42.6) | 0.005 |
| BMI, kg/m² | 23.9 ± 2.9 | 24.0 ± 3.0 | 0.021 |
| Current smoking | 1,119 (18.0) | 1,131 (18.2) | 0.005 |
| Current alcohol consumption | 1,148 (18.5) | 1,156 (18.6) | 0.003 |

Note: Propensity scores were estimated using age, sex, BMI, current smoking, and current alcohol consumption. Medical radiation workers and healthcare controls were matched 1:1 using nearest-neighbor matching without replacement and a caliper width of 0.2 SD of the logit of the propensity score.

**Supplementary Table S7. Sensitivity analyses of the association between TyG and structural chromosome aberration count among medical radiation workers**

| **Analytical modifications** | **Adjusted IRR (95% CI)** | ***P* value** |
| --- | --- | --- |
| Panel A: Excluding participants with self-reported diabetes or current lipid-lowering therapy (*n* = 22,284) | | |
| Q1 (<8.33) | 1.00 (Reference) | — |
| Q2 (8.33 to <8.63) | 1.06 (0.88–1.28) | 0.541 |
| Q3 (8.63 to <8.92) | 1.21 (1.01–1.46) | 0.043 |
| Q4 (≥8.92) | 1.45 (1.21–1.74) | <0.001 |
| *P* for trend |  | 0.001 |
| TyG, per 1-SD increase | 1.13 (1.07–1.21) | <0.001 |
| Panel B: Modeling BMI as a categorical variable (<24, 24-27.9, and ≥28 kg/m^2^) | | |
| Q1 (<8.33) | 1.00 (Reference) | — |
| Q2 (8.33 to <8.63) | 1.09 (0.91–1.32) | 0.352 |
| Q3 (8.63 to <8.92) | 1.26 (1.03–1.53) | 0.021 |
| Q4 (≥8.92) | 1.51 (1.26–1.81) | <0.001 |
| *P* for trend |  | <0.001 |
| TyG, per 1-SD increase | 1.14 (1.08–1.21) | <0.001 |
| Panel C: Zero-inflated negative binomial regression | | |
| Q1 (<8.33) | 1.00 (Reference) | — |
| Q2 (8.33 to <8.63) | 1.08 (0.90–1.31) | 0.384 |
| Q3 (8.63 to <8.92) | 1.25 (1.04–1.51) | 0.017 |
| Q4 (≥8.92) | 1.48 (1.23–1.78) | <0.001 |
| *P* for trend |  | <0.001 |
| TyG, per 1-SD increase | 1.14 (1.08–1.21) | <0.001 |

Note: Models were adjusted for age, sex, BMI, current smoking, current alcohol consumption, occupational category, and duration of radiation-related employment; BMI was modeled categorically in Panel B. Original TyG quartile boundaries were retained in Panel A. For Panel C, the reported IRRs were derived from the conditional count component of the zero-inflated negative binomial model.

**Supplementary Table S8. Distribution of participants excluded because of self-reported diabetes or current lipid-lowering therapy across predefined TyG quartiles**

| **Predefined TyG quartile** | **TyG boundary** | **Excluded participants, n** | **Distribution among excluded participants, %** | **Exclusion prevalence within quartile, %** |
| --- | --- | --- | --- | --- |
| Q1 | <8.33 | 45 | 4.0 | 0.8 |
| Q2 | 8.33 to <8.63 | 125 | 11.1 | 2.1 |
| Q3 | 8.63 to <8.92 | 310 | 27.6 | 5.3 |
| Q4 | ≥8.92 | 644 | 57.3 | 11.0 |
| Total | — | 1,124 | 100.0 | 4.8 |

Note: Exclusions were based on self-reported diabetes or current lipid-lowering therapy. Quartile boundaries were retained from the original medical radiation-worker population.

**Supplementary Table S9. Sensitivity analysis additionally adjusting for duration of radiation-related employment in the stratified dosimetry substudy**

| **Model** | **Adjusted IRR per 1-SD increase in TyG (95% CI)** |
| --- | --- |
| Primary dosimetry model | 1.27 (1.14–1.42) |
| Additionally adjusted for duration of radiation-related employment | 1.26 (1.13–1.41) |

Note: The primary model was adjusted for recorded cumulative occupational dose, occupational category, age, sex, BMI, current smoking, and current alcohol consumption. Recorded cumulative occupational dose was modeled continuously; 1 SD of TyG was 0.45.

**Supplementary Table S10. Association between TyG and total structural chromosome aberration count by occupational category in the stratified dosimetry substudy**

| **Occupational category** | **n** | **Participants with ≥1 structural chromosome aberration, n (%)** | **Adjusted IRR per 1-SD increase in TyG (95% CI)** |
| --- | --- | --- | --- |
| Diagnostic radiology | 500 | 25 (5.0%) | 1.21 (0.95–1.54) |
| Nuclear medicine | 500 | 45 (9.0%) | 1.25 (1.05–1.49) |
| Interventional radiology | 500 | 62 (12.4%) | 1.31 (1.11–1.55) |

Note: Models were adjusted for recorded cumulative occupational dose, age, sex, BMI, current smoking, and current alcohol consumption. Recorded cumulative occupational dose was modeled continuously; 1 SD of TyG was 0.45. *P* for interaction across occupational categories = 0.628.
